# Expression of recombinant staphylokinase in the methylotrophic yeast *Hansenula polymorpha*

**DOI:** 10.1186/1472-6750-12-96

**Published:** 2012-12-19

**Authors:** Manal Moussa, Mahmoud Ibrahim, Maria El Ghazaly, Jan Rohde, Stefan Gnoth, Andreas Anton, Frank Kensy, Frank Mueller

**Affiliations:** 1 Minapharm Pharmaceuticals, Cairo, Egypt; 2 Scil Proteins, Halle, Germany; 3 m2p-Labs, Baesweiler, Germany

**Keywords:** Staphylokinase, *Hansenula polymorpha*, Recombinant protein, Fermentation, Scale-up, HTP

## Abstract

**Background:**

Currently, the two most commonly used fibrinolytic agents in thrombolytic therapy are recombinant tissue plasminogen activator (rt-PA) and streptokinase (SK). Whereas SK has the advantage of substantially lower costs when compared to other agents, it is less effective than either rt-PA or related variants, has significant allergenic potential, lacks fibrin selectivity and causes transient hypotensive effects in high dosing schedules. Therefore, development of an alternative fibrinolytic agent having superior efficacy to SK, approaching that of rt-PA, together with a similar or enhanced safety profile and advantageous cost-benefit ratio, would be of substantial importance. Pre-clinical data suggest that the novel fibrinolytic recombinant staphylokinase (rSAK), or related rSAK variants, could be candidates for such development. However, since an efficient expression system for rSAK is still lacking, it has not yet been fully developed or evaluated for clinical purposes. This study’s goal was development of an efficient fermentation process for the production of a modified, non-glycosylated, biologically active rSAK, namely rSAK-2, using the well-established single cell yeast *Hansenula polymorpha* expression system.

**Results:**

The development of an efficient large scale (80 L) *Hansenula polymorpha* fermentation process of short duration for rSAK-2 production is described. It evolved from an initial 1mL HTP methodology by successive scale-up over almost 5 orders of magnitude and improvement steps, including the optimization of critical process parameters (e.g. temperature, pH, feeding strategy, medium composition, etc.). Potential glycosylation of rSAK-2 was successfully suppressed through amino acid substitution within its only N-acetyl glycosylation motif. Expression at high yields (≥ 1g rSAK-2/L cell culture broth) of biologically active rSAK-2 of expected molecular weight was achieved.

**Conclusion:**

The optimized production process described for rSAK-2 in *Hansenula polymorpha* provides an excellent, economically superior, manufacturing platform for a promising therapeutic fibrinolytic agent.

## Background

Staphylokinase (SAK), a protein produced by certain *Staphylococcus aureus (S. aureus)* strains, was found to possess pro-fibrinolytic properties almost 6 decades ago [1-3]. In comparison with streptokinase (SK), recombinant wild-type staphylokinase (wt-SAK) has shown higher fibrinolytic efficacy as well as lower fibrinogenolytic effects [4]. However, as a heterologous (bacterial) protein, wt-SAK produces immunogenic effects in animals that compromise its effectiveness on repeated administration and thus, potentially, its efficacy in clinical applications [5].

Laroche et al. [6] showed that wt-SAK immunogenicity can be significantly reduced by site-directed mutagenesis to eliminate antigenic epitopes present within the protein sequence, while conserving its activity. Since then, several studies have investigated the immunogenic and activity properties of numerous SAK variants. In pre-clinical trials, a sequence optimized SAK variant named THR174, developed at ThromboGenics NV http://www.thrombogenics.com; [7], showed improved efficacy and safety profiles as well as significantly reduced immunogenicity compared with SK and other SAK variants.

### Expression system

SAK variants are routinely produced in *Escherichia coli (E. coli)*[8] and *Bacillus subtilis (B. subtilis)*[9] expression systems. The methylotrophic yeast *Hansenula polymorpha (H. polymorpha)* provides an alternative expression system and has already been successfully developed for several commercial production processes, e.g. HBsAg vaccine particles, Interferon-α2a and Hirudin [10-12]. *H. polymorpha* is able to utilize methanol as sole carbon source, resulting in over-expression of ‘methanol utilization’ enzymes, namely methanol oxidase (MOX), dihydroxyacetone synthase (DHAS) and formate dehydrogenase (FMD). Heterologous DNA is readily and stably integrated into the *H. polymorpha* genome using routinely applied techniques to achieve high copy, heterologous gene numbers. The strongly inducible promoters of the *MOX* and *FMD* genes are commonly used as regulatory components for enhanced heterologous gene expression, by which correspondingly high protein concentrations, up to 70% of total soluble cell protein content, are attained [11]. Furthermore, the *S. cerevisiae*-derived MFα1 leader sequence can be used to target the heterologous protein produced in *H. polymorpha* for secretion, allowing its easy recovery from the fermentation broth [13-17].

The combination of stable heterologous gene expression, synthesis and secretion of high concentrations of recombinant proteins, the advantageous characteristics of the *H. polymorpha* expression system, would appear to provide the means for developing a production process for a potentially clinically superior rSAK variant. Therefore, the aim of this study was the development of an efficient *H. polymorpha* fermentation process in stirred tank bioreactors to yield high concentrations of the rSAK variant, THR174 [7]. The full upstream development, starting with gene design, cloning and media optimization at the micro-scale, up to large scale fermentations in stirred tank bioreactors is described.

## Results and discussion

### Strain selection

**Figure 1:**
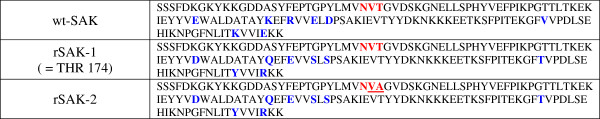
**Amino acid sequences of wt-SAK and SAK-variants [rSAK-1 (= THR 174) and rSAK-2]. **The amino acid sequence of each of the different SAK molecules, highlighting the differences in their primary structures (blue), is shown. Red: Potential N-linked glycosylation-site motif. Red underlined: Amino acid exchange Thr-30 → Ala-30.

**Figure 2:**
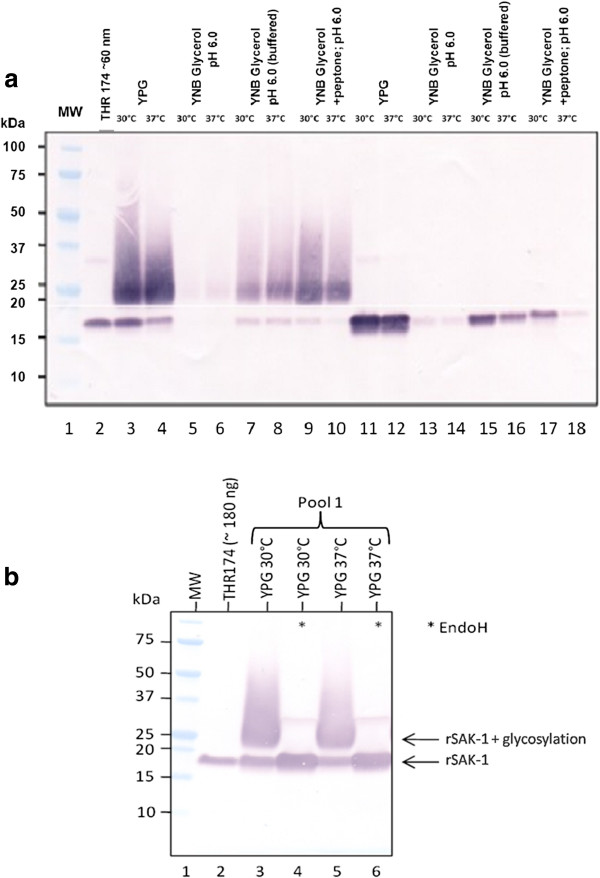
**a. Western blot analysis of supernatants from rSAK producing strains. **Lane 1 THR 174 ~ 60ng; RB11/pFPMT Mfα^+ ^- rSAK-1 (lane 2 – 10) and RB11/pFPMT Mfα^+ ^- rSAK-2 (lane 11–18); 7 μL loaded. **b**: Glycosylation evidence for SAK: SDS-PAGE analysis of de-repression supernatants. (Pool 1) was de-repressed in YP 2% glycerol for 40 h at 30°C and 37°C. Pooled supernatants of pool 1 were treated with EndoH (lane 4 and 6). Supernatants without EndoH treatment (lane 3 and 5) have been handled with the same EndoH buffers but omitting EndoH. After EndoH treatment, samples were prepared for SDS PAGE and 21 μL each was loaded on a Criterion 4-20% precast gradient gel. Supernatants have been finally examined in a Western blot analysis for presence of SAK. SAK protein was detected by using alkaline phosphatase-conjugated mouse anti-SAK antibody. Approximately 180 ng of THR174 served as positive control.

Initial trials for the correct protein expression productivity of rSAK-1 and rSAK-2 (see Figure 1) transformants (strains) were performed in shake flasks using YPG medium. As shown in Western blots of culture supernatants from the rSAK-2 strain (Figure 2a), a single well-defined band at around 16 kDa that migrated parallel to that of *E. coli*-produced rSAK-1 (ThromboGenics NV) is present. The rSAK-1 strain, however, produced an additional ‘smear-like’ band between 20 kDa and 25 kDa, indicating glycosylated rSAK-1.

Endoglycosidase H treatment of supernatant from the rSAK-1 strain’s fermentation led to removal of the smear-like band, while the well-defined band at 16 kDa, corresponding to correctly processed, non-glycosylated rSAK-1, significantly increased in intensity (Figure 2b). This provided evidence that the (*H*. *polymorpha*) rSAK-1 strain secretes both glycosylated and non-glycosylated rSAK-1, while the rSAK-2 strain secretes only non-glycosylated rSAK-2 (Figure 2b).

Since glycosylated rSAK-1 is known to have substantially reduced enzymatic activity and was predicted to pose various difficulties for downstream processing and product characterization [13,17], the rSAK-2 strain secreting only non-glycosylated rSAK-2 was chosen for further process development.

A series of different cultivation techniques were applied during the upstream development. Cultures ranging from 1 mL volume to 80 L volume scale were investigated under fully and/or partially controlled conditions. An overview of applied cultivation systems and their usage in this study is provided in Table 1.

**Table 1:** Overview of applied cultivation systems and their usage described herein

**Cultivation scale [L]**	**Cultivation dimension**	**Cultivation system**	**Optimization parameter**	**Max. rSAK-2 conc. [mg/L]**	**Cell density [OD**_**600**_**]**	**Time [h]**	**Specific productivity [mg/OD**_**600**_**/h]**	**Media**
0.001	Microtiter plate	Biolector	pH – media – feeding strategy	90	n.d.	n.d.	n.d.	SYN6 + wheat peptone
0.1	Shake flask (batch)	-	Media	31	35	72	0.012	SYN6.4
	Shake flask (fed-batch)	-	Feed composition - media	200	130	48	0.032	SYN6.46d
0.300	Parallel fermenter system	DASGIP	pH – media –feeding strategy/composition	423	291	48	0.030	SYN6.46d
2	Fermenter	Sartorius Biostat B Plus 3L	pH – feed rate/composition – fermentation duration	1212	263	75	0.061	SYN6.46d
8	Fermenter	Sartorius Biostat C 15L	Upscale	1081	340	70	0.045	SYN6.46d
80	Fermenter	Sartorius Biostat C 150L	Upscale	1109	370	70	0.043	SYN6.46d

### Fermentation process development and scale-up

The upstream process development was started in the microscale BioLector® and small scale shake flask systems. The process was further developed in 300 mL and 2 L scale bioreactors and finalized with a linear scale-up to 8 L and 80 L.

### Microscale BioLector®

#### pH optimization

Optimization of rSAK-2 expression was commenced at the micro-scale fermentation level (1 mL) by studying biomass growth and protein expression ratio in SYN6 medium buffered to different pH values. We found that the cells grew best at pH 4, but that rSAK-2 expression was only detected between pH 6 and 7. At pH values higher than 7, biomass growth was significantly inhibited (data not shown). In addition, published data on pH stability of SAK [18] indicated that it is most stable between pH 6 and 7. Therefore, a constant pH of 6.5 was chosen for all following micro-scale experiments.

### Medium optimization

**Figure 3:**
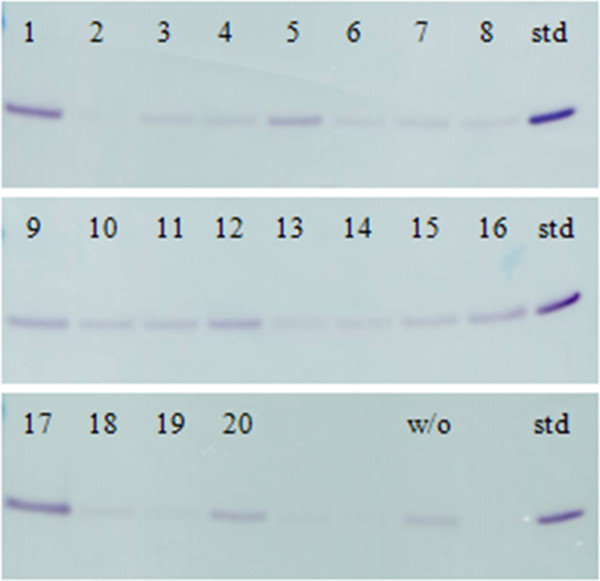
**Western blot analysis of rSAK-2, expressed in SYN6 medium supplemented with 20 different plant peptones and a comparative fermentation without peptone. **Results from a 1 ml micro scale batch fermentation in the BioLector® system (5 μL supernatant loaded). Final lanes loaded with THR-174 (~109 ng). The most prominent SAK-2 signals appeared for vegetable peptone (1) and wheat peptone (17).

**Figure 4:**
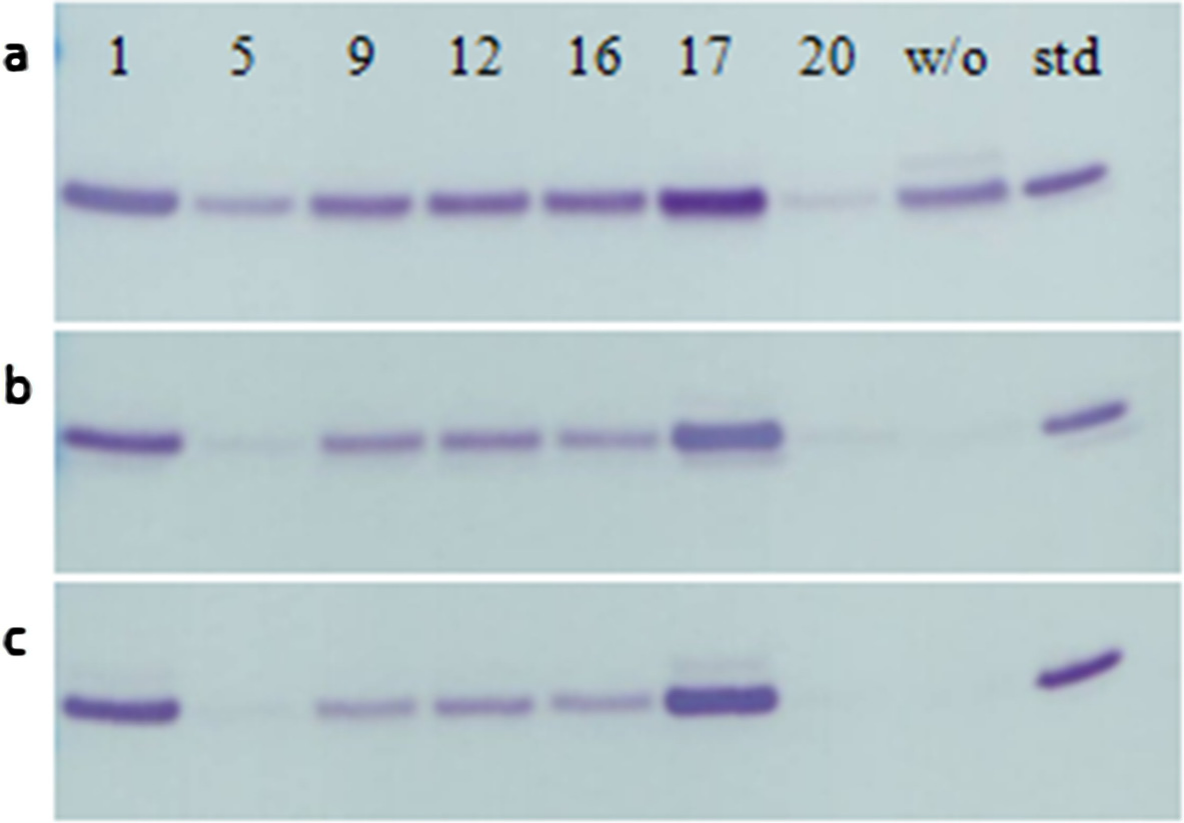
**Western Blot analysis of rSAK-2, expressed in SYN6 medium supplemented with different plant peptones and a comparative fermentation without peptone (5 μL supernatant loaded). **Sampled: **a**) at the end of exponential phase, **b**) after 2 hours and **c**) after 5 hours after the entry in stationary phase. Highest results from a 1 mL micro scale batch fermentation in the BioLector® system were attained with wheat peptone (17) followed by vegetable peptone (1). Final lanes were loaded with THR-174 (~109 ng).

The effect of addition of 20 different plant peptones to SYN6 medium on rSAK-2 expression was assessed (Figure 3). From this survey, 7 promising peptone candidates were picked for further kinetic investigations. For this purpose, 3 different phases of sampling of the fermentation process were indicated by the online process data: 1. sampling - end of exponential phase; 2. sampling - 2 hours after entry into stationary phase and 3. sampling - 5 hours after entry into stationary phase (Figure 4).

In general, the secreted protein in the cell supernatant is stabilized by peptones, though not all peptones have a positive effect on protein expression. We found fermentations with wheat peptone yielded highest, stabilized, concentrations of rSAK-2. In comparison, other peptones, or just ‘no peptone supplementation (w/o)’, resulted in degradation of rSAK-2 over time (data not shown). Consequently, wheat peptone was chosen for all further experiments.

### Feed strategy

**Figure 5:**
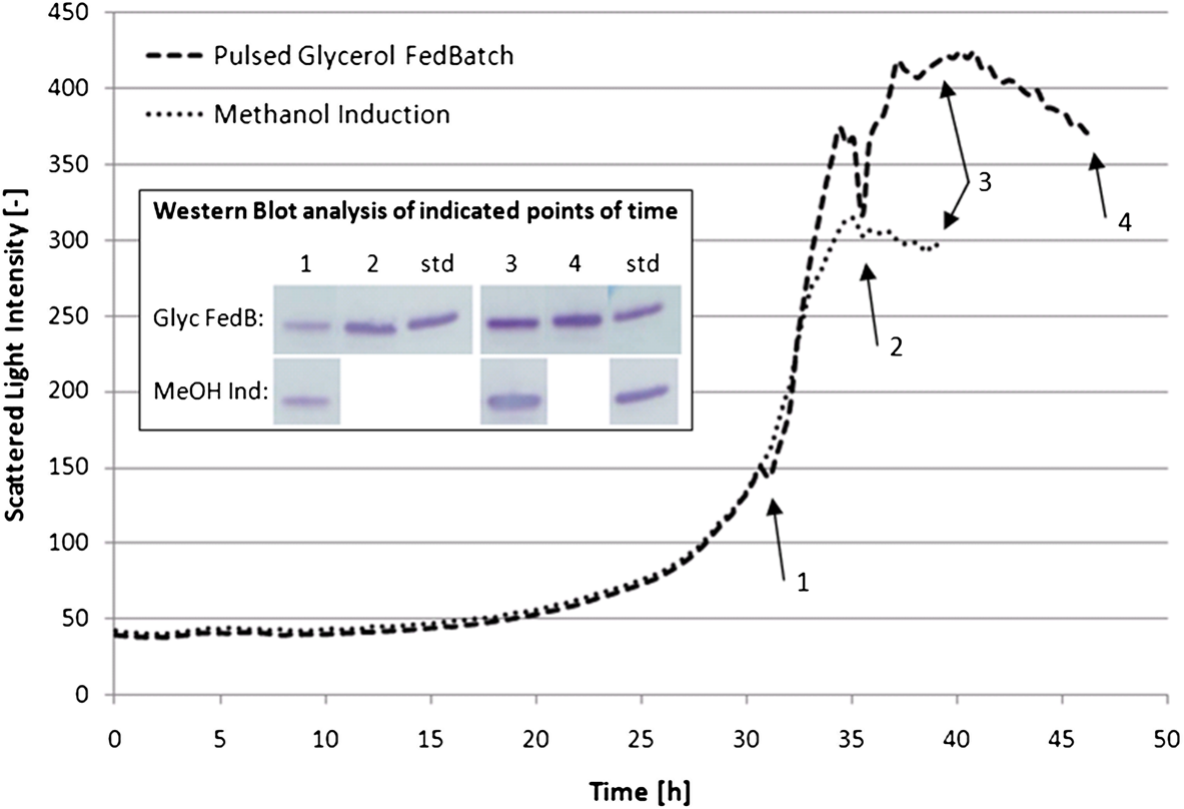
**Biomass growth over time of a glycerol fed-batch culture and a methanol induced culture, respectively, grown in SYN6 media. **The Fed-Batch culture was pulsed twice at t = 1 and 2 with a mixture of 10 g/L glycerol and 10 g/L peptone (final concentration). At four points of time (1–4) a sample was taken. For the other culture, methanol was given once (1) at a final concentration of 10 g/L. Samples were taken at t = 1 and 3. Fermentation was carried out at 1mL scale in the BioLector® system. Samples were analysed in a Western Blot for rSAK-2 expression (5 μL supernatant loaded). Standards (std) are drawn for the individual gels. Final lanes were loaded with THR-174 (~109 ng).

In further micro-scale experiments using SYN6 medium supplemented with 10 g/L wheat peptone, two feed strategies were assessed in parallel. In the first, a ‘pulsed’ glycerol-peptone fed-batch fermentation was carried out. The feed solution was pulsed manually at the end of the exponential growth phase when the dissolved oxygen (DO) signal increased. This was done twice during fermentation at the time points indicated (Figure 5). At time points 1 to 4, samples were taken. In the second, a culture was induced with methanol at time point 1. The methanol-induced culture received no additional nutrients during fermentation and samples were taken at time points 1 and 3.

RP-HPLC analysis of samples from the two feeding strategies revealed a 1.5-fold higher rSAK-2 protein concentration for the methanol-induced culture versus the ‘pulsed’ glycerol-peptone fed-batch (90 mg/L versus 60 mg/L, respectively). Therefore, it was decided to base further fermentation strategy experiments on the methanol induction procedure.

### Small scale shake flasks

#### Temperature

Shake flask batch fermentations comparing temperatures of 30°C and 37°C were performed. The cells consistently showed higher growth at 30°C compared to 37°C. The maximum OD_600 _ reached at 30°C was around 40, whereas at 37°C it was around 30. While this slightly higher cell growth rate at 30°C was not significant, a fermentation temperature of 30°C was set for all following investigations.

### Media optimization

In small scale shake flask fermentations performed in parallel with micro-scale experiments, the effect on rSAK-2 concentration of adding yeast extract to SYN6 medium was assessed. This was carried out together with varying combinations and concentrations of the medium’s components, including wheat peptone. Twenty seven different variations of medium supplements were tested in batch fermentations without feed addition. The supplemented medium yielding the highest rSAK-2 concentration, 31 mg/L as determined by RP-HPLC, was selected for further optimization. The ‘in-house developed’ medium named SYN6.4 contained 4% w/v wheat peptone and 3% w/v yeast extract and had modified concentrations of 1% v/v each of the four supplement solutions (normally 2% v/v CaCl_2_, 0.5% v/v vitamins, 0.5% v/v micro elements and 0.5% v/v trace elements), which are components of ‘classical’ SYN6 medium.

Further optimization of SYN6.4 medium was performed in fed-batch fermentations at the shake flask scale. Our approach was to remove one or more of the defined components of SYN6.4 medium in each flask in order to discover “inessential” constituents and those that are limiting for rSAK-2 expression. Interestingly, it was found that the highest rSAK-2 yield of 200 mg/L occurred when all defined trace elements and vitamins together with EDTA were removed from the medium. We called this medium SYN6.46d (Table 2).

**Table 2:** Overview of SYN 6.[X] media compositions

**Media Components**	**SYN 6**	**SYN 6.4**	**SYN 6.46d**
Glycerol	2% w/v	2% w/v	2% w/v
Salt mix solution	10% v/v	5% v/v	5% v/v
Wheat peptone	-	4% w/v	4% w/v
Yeast extract	-	3% w/v	3% w/v
CaCl_2 _solution	1% v/v	2% v/v	2% v/v
Vitamins solution	1% v/v	0.5 % v/v	-
Micro elements solution	1% v/v	0.5 % v/v	0.5 % v/v
Trace elements Solution	1% v/v	0.5 % v/v	-

### Feed strategy optimization

The feed strategy of methanol induction developed in the microscale BioLector® was tested and further optimized in fed-batch shake flasks. In addition to methanol, glycerol was added to the feed solution (FS) to maintain cell growth during the initial period of the fed-batch phase. We also added wheat peptone and yeast extract to the FS to replenish the nutrients consumed during growth. The feed strategy with these 4 supplements led to a significant increase in expressed r-SAK-2 (Table 1). Different amounts of the feed solution supplements were then tested in the 300 mL bioreactor.

#### 300 mL scale bioreactor

##### pH optimization

Stirred bioreactor fermentations permitted implementation of a wider pH range. Due to the ability to control the pH, it was decided to keep it constant during the biomass growth phase at 5.5 to maintain a reasonable growth rate. Then, without significantly limiting biomass growth, to increase the pH gradually during the fed-batch phase to 6.3 to promote increased rSAK-2 expression, secretion and stability.

##### Feed strategy optimization

Using the in-house developed medium SYN6.46d for fermentation, we performed fed-batch cultivations in 300 mL DASGIP bioreactors in order to ascertain the optimum composition of the ‘feeding solution’ (FS).

Two different FS were compared. The first FS consisted of 20% yeast extract (w/v), 10% peptone (w/v), 5% glycerol (w/v) and 10% methanol (w/v) (called FEED_I). The second FS had increased peptone and glycerol concentrations and consisted of 20% peptone (w/v), 10% glycerol (w/v), 20% yeast extract (w/v) and 10% methanol (w/v) (called FEED_II). Feeding was done in lots of 20 mL FS every 5 hours and was automated via the DASGIP bioreactor’s pump module. RP-HPLC analysis of culture supernatants gave concentrations of 149 and 213 mg rSAK-2/L for FEED_I and FEED_II, respectively. Their specific productivities were 0.018 and 0.027 mg/OD_600_/h respectively. Therefore, FEED_II was chosen for all subsequent experiments.

From previous experience with *H. polymorpha*, it was known that different feeding strategies can significantly impact on heterologous protein production. In the 300 mL DASGIP bioreactor, we compared the ‘batch-wise mode’ feeding with a 20 mL FS [FEED_II] every 5 hours against a ‘constant feeding mode’ with a rate of 4 mL FS/hour, as well as a pO_2_-driven feeding procedure. The constant feeding mode of 4 mL FS/hour gave the highest rSAK-2 expression at 423 mg/L, significantly more than the other two feeding strategies; 304 mg/L with batch-wise feed and 295 mg/L with pO_2_-driven feed.

#### Scale-up and optimizations in 2 L bioreactors

While attaining rSAK-2 concentrations of around 400 mg/L at the 300 mL fermentation scale over 48 hours’ duration, it was necessary to reproduce this yield in a scaled-up fermentation of 2 L. For scale-up trials, the conditions that were found best during our previous experiments at the 300 mL scale were used. These included the use of the in-house developed medium SYN6.46d, the ‘constant feeding mode’ and the optimal FS composition. However, aeration, pH and stirring had to be adjusted as described in the methods section. The feeding rate was set to 18 mL/hour to match the larger cultivation volume.

Under such optimized conditions, trials at the 2 L scale resulted in rSAK-2 yields of up to 480 mg/L.

**Figure 6:**
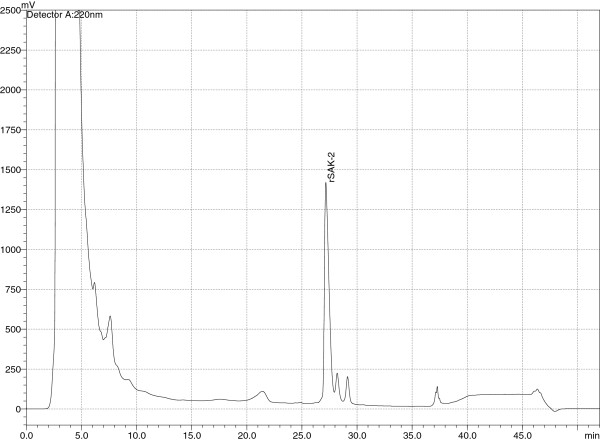
Typical RP-HPLC profile of a final rSAK-2 fermentation supernatant sample at 73.5 hours of cultivation.

As sampling at different fermentation times has shown increasing rSAK-2 expression, fermentation to a total of 70–73 hours with constant feeding over 50 hours was evaluated. Due to the requirement for increased volume of feed solution, the initial volume of medium had to be reduced from 1.4 to 1.2 L, the feed rate re-adjusted to 20 mL/hour and the FS composition re-adjusted to 20% peptone (w/v), 22% glycerol (w/v), 20% yeast extract (w/v) and 15% methanol (w/v). An rSAK-2 concentration of 1212 mg/L was attained under these final optimized conditions. This production level was reproducible and consistent in several fermentations (data not shown). Figure 6 shows a typical RP-HPLC profile for an rSAK-2 containing supernatant at the end of fermentation.

#### Scale-up to 8 L and 80 L bioreactors

**Figure 7:**
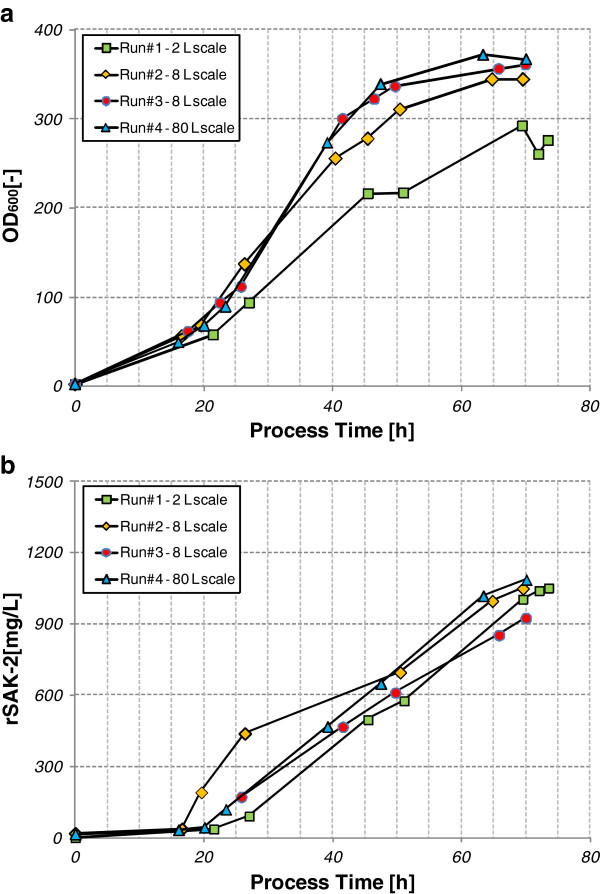
**a. Overlay of OD**_**60 **_**values for rSAK-2 fermentations at different bioreactor scales. b: Overlay of rSAK-2 titers for fermentations at different bioreactor scales.**

The final optimized conditions for the 2 L scale were successfully transferred to the 8L and 80L scales (see Figures 7a and b). To show the reproducibility of the fermentation process at these volumes, two trial fermentations were performed at the 8 L scale and one at the 80L scale, with the following results: (Table 3).

**Table 3:** rSAK-2 concentrations attained at the 8L and 80L scale

**Trial**	**Scale**	**rSAK-2 concentration [mg/L]**
1	8 L	1081
2	8 L	925
3	80 L	1109

Activity measurement by the plasminogen activation assay (PAA) for fermentation supernatant samples gave a specific activity of rSAK-2 within the range of that of the recombinant SAK (STAR) reference standard 94/718 (obtained from The National Institute for Biological Standards and Controls (NIBSC), UK). Specific activity results are shown in Table 4 and take into consideration that the STAR reference standard is a purified lyophilized material stabilized in an albumin containing phosphate buffer.

**Table 4:** Summary of specific biological activities for rSAK-2 from crude fermentation supernantant samples after harvest compared to the SAK reference standard from NIBSC

**Scale/Sample**		**Measurement number**	**Concentration by PAA [μg/ml]**	**Activity by PAA [IU/ml]**	**Specific activity [IU/mg]**
**80 L scale**	**Batch 1**	1	883.87	55.59	58.302
		2	783.87		
	**Batch 2**	1	649.03	42.32	58.376
		2	620.65		
	**Batch 3**	1	816.13	52.42	56.232
		2	756.45		
**2 L** **scale**		1	999.19	60.73	58.446
		2	822.58		
**SAK STAR NIBSC 94/718 (1 IU = 15 μg as described in datasheet)**		Theoretical value	15	1	66.667

That cell growth, as measured OD_600 _values, at the 8 L and 80 L bioreactor scale was higher than at the 2 L scale can be explained by the higher power input in stirring and aeration in the larger bioreactors. The rSAK-2 concentrations attained in the 2 L, 8 L and 80 L bioreactors were within the same range. Taking into account the differences in cell growth, the specific productivity at larger scales was lower than in the 2 L bioreactor (Table 1).

Development and optimization of the *H. polymorpha* fermentation process for rSAK-2 production was considered final at this stage.

## Conclusion/discussion

In the past, production of rSAK or its several molecular variants has been carried out in a number of expression systems, including *E. coli*, *B. subtilis* and *P.pastoris*. Table 5 (below) gives an overview of published data.

**Table 5:** Overview of different Staphylokinase expression systems published to date

**No**	**Expression system**	**SAK Productivity [mg/L**_**broth**_**]**	**Cultivation system**	**Volume [L]**	**SAK - glycosylation [Yes/No]**	**Reference**
1	*E. coli*	> 50	(not defined)	2.7	No	[8]
2	*E. coli*	> 175	Fermenter	10-15	No	[19]
3a	*B. subtilis*	140	Shake flask	0.25	No	[20]
3b	*B. subtilis*	337	Fermenter	2	No	[20]
4a	*P.pastoris*	110	Fermenter	< 5	Partially yes	[13]
4b	*E. coli*	300-400	Shake flask	0.1	No	[13]
5	*E. coli*	2800	Fermenter	2	No	[21]
6	*P.pastoris*	~ 1000	Shake flask (w/o -tunicamycin)	0.05	Yes	[17]
7	*P.pastoris*		Shake flask (w - tunicamycin)	0.05	No	[17]
8	*H. polymorpha*	~ 1100	Fermenter	80	No	-

In addition, in this study, we have shown the methylotrophic yeast *H. polymorpha* is highly suitable for the expression of a sequence-optimized rSAK variant, rSAK-2, of reduced immunogenic potential.

Initial kinetic fermentation and medium composition data obtained from high throughput microreactors at a 1 mL scale provided a very reliable and robust basis for successfully scaling the laboratory process for rSAK-2 production to large scale fermenters (stirred tank bioreactors). During upstream process development and up-scaling of fermentations over almost 5 orders of magnitude, we were able to systematically optimize the fermentation process in a stepwise manner to attain high levels of rSAK-2 expression and secretion. Further optimization of the process included the use of an in-house developed medium containing both undefined supplements (peptone and yeast extract) and defined components (components of SYN6 medium). We showed the use of this medium resulted in significantly higher yields of rSAK2 compared to “classical” unmodified SYN6 growth medium, which has often been used for *H. polymorpha* fermentations [11].

Thus, a simple, relatively fast, robust and reproducible fermentation protocol was developed that, on implementation at the large scale, yielded concentrations greater than 1g/L of the intact, biologically active, rSAK-2 protein. In essence, this fermentation protocol consists of only two phases: a glycerol batch phase and a methanol/glycerol fed-batch phase. It therefore eliminates the need for a glycerol de-repression fed-batch phase before the methanol/glycerol induction fed-batch phase, as is usual for *H. polymorpha *fermentations [11]. Thereby fermentation time was considerably shortened, though relatively high cell densities and very high rSAK-2 protein expression levels were still maintained.

Compared to other expression systems, as listed in Table 5, *H. polymorpha* has several key advantages:

1. With a simple fermentation protocol, the secreted protein, rSAK-2, accumulates in high concentration in the cell culture supernatant. When compared to unwieldy extraction from inclusion bodies, typical for *E. coli,* secretion leads to simplification of subsequent downstream processing, and thus has a positive impact on the overall process’ economics.

2. The intended product, rSAK-2, is secreted as a biologically active, non-glycosylated protein. Compared with expression in *P. pastoris*, our process does not require addition of tunicamycin for the suppression of glycosylation [17].

3. Compared with expression in *B. subtilis* or *E. coli*, where micro-heterogeneity – in particular, N-terminal truncated SAK-variants lacking 6 – 11 N-terminal amino acids – is of major concern [8,20], rSAK-2 is secreted in a highly homogeneous and relatively pure form (Figure 6), thus simplifying its final purification.

4. Compared with other expression systems, expression in *H. polymorpha* lends itself to the implementation of a relatively simple final process design that permits an easy scale-up and incorporates a high degree of process’ robustness.

Altogether, our optimized process resulting in high concentrations of target protein, namely rSAK-2, provides an excellent platform to initialize the downstream process development of this promising fibrinolyic agent for future clinical evaluation. We conclude our optimized *H. polymorpha*-based upstream process approach offers a significantly superior rSAK production strategy than in other expression systems that are currently being trialed. Our production strategy and findings should encourage the further development and pre-clinical evaluation of rSAK-2 (or other rSAK variants) as a potential replacement or alternative therapeutic fibrinolytic agent for those therapeutics, e.g., SK and rt-PA, already in the field.

## Methods

### Gene constructs and expression plasmids

THR174 (called in this paper rSAK-1) has one possible N-glycosylation recognition motif, comprised by amino acids in positions 28 to 30 (Asn-Val-Thr), with Asn-28 representing the actual carbohydrate attachment site. Since glycosylated variants of SAK are known to be of diminished activity [17,18,22] and *H. polymorpha* is known to be able to perform N-glycosylation, amino acid Thr-30 was substituted by an alanine for expression of SAK in this yeast, resulting in a variant called rSAK-2, which lacks the motif for glycosylation (see Figure 1).

The rSAK-1 and rSAK-2 genes were assembled by *de novo* synthesis (GeneArt AG), streamlined for transcription and adapted to *H. polymorpha* codon usage. Gene synthesis also included the addition of flanking restriction sites required for convenient subcloning into the expression vector pFPMTMFα^+^. This vector bears the *S. cerevisiae* URA3 selectable marker and also contains a sequence encoding the *S. cerevisiae* preproMFα1 secretion leader element between the FMD promoter and the MOX terminator elements. Cloning into a unique HindIII site engineered into the leader sequence created an in-frame fusion of the leader to the target gene sequence.

### Generation of *H. polymorpha* strains secreting rSAK

The expression plasmids were transformed into the uracil-auxotrophic *H. polymorpha* strain RB11 by electroporation. Ura^+^ transformants were selected on YNB/glucose plates (0.17% YNB w/o amino acids and 0.5% ammonium sulfate/2% glucose containing 2% Bacto agar) [23,24]. Single transformant colonies were cultured in 3 mL cultivation tubes (shake flasks) in sequential cycles of growth in liquid medium (40 hours at 180 rpm at 37°C), first in selective medium (YNB/glucose) to induce plasmid multiplication associated with spontaneous genomic integration, and then in non-selective medium (YPD; 2% Bacto peptone/1% yeast extract/2% glucose) to eliminate any remaining unintegrated plasmids. This 'passaging' procedure has been proven to yield mitotically stable integrants containing multiple copies of the transformed plasmid [24].

The integrant cell lines were screened for rSAK expression after cultivation in YPG medium (2% Bacto peptone/1% yeast extract/1% glycerol). The presence of glycerol as the sole carbon source resulted in de-repression of the FMD promoter. After 40 hours of growth at 180 rpm at 37°C, the cell-free culture supernatants were analyzed for the presence of rSAK protein by immunoblotting with a mouse monoclonal antibody developed against rSAK-1 (provided by ThromboGenics NV). High-level secreting integrant lines were selected and individualized by plating for single colonies. Ten randomly selected colonies, each representing a genetically pure clone, from each integrant line were re-screened as described above to identify the most efficiently secreting strains (data not shown). Strain rSAK-2 #1/4 was chosen for further analyses described in this study.

### Fermentation process development and scale Up

#### Microscale BioLector®

Initial growth medium optimization and pH screening were performed with the BioLector® (m2p-labs GmbH, Aachen, Germany), a system that allows for intensive studies of 48 parallel fermentation processes at 1 mL scale in microtiter plates. For these experiments, a specially shaped Flowerplate® was used that simultaneously provides elevated oxygen transfer rates (OTR) and high information content. Biomass concentration, fluorescence, pH and dissolved oxygen (DO) can be measured for each individual well by non-invasive optical measurements.

For growth medium optimization, the SYN6 medium [11] was chosen as the ‘basic formulation’ and then modified in several ways. Fermentations at different pHs in the range from 4–8, operated in 0.5 steps, were first performed. Based on the outcome of this evaluation, a panel of 20 different plant peptones (Sigma, Art.-No. 11577) was used at 10 g/L for screening of positive effects on rSAK-2 expression. Lastly, two different expression strategies were investigated in the BioLector®, namely a pulsed glycerol-peptone fed-batch and a methanol-induced culture. Glycerol, peptone, as well as methanol, were each dosed at 10 g/L (final concentration). A 100 mM phosphate buffer was used to maintain the pH value at 6.5.

All small scale experiments were inoculated with overnight grown YPD shake flask cultures at an initial OD_600 _of 1.0. Cultivation in the BioLector® was carried out at 30°C and shake frequencies of 1000–1300 rpm (3 mm diameter), thus eliminating oxygen deprivation. Process parameters were measured in a cycle of 10 minutes.

#### Small scale shake flasks

Batch shake flask studies were performed firstly by conducting fermentations at the temperatures 30°C or 37°C. Further media optimization trials in the shake flask scale followed the temperature comparison. An overnight grown YPG culture was used to inoculate shake flask cultivations in 500 mL baffled flasks with a working volume of 100 mL: different in-house developed medium variants based on SYN6 medium were tested. All media were supplemented with 2% w/v glycerol as carbon source. Cultures were shaken in an incubator at 30°C and 180 rpm for 72 hours. Samples were taken every 24 hours for OD_600 _ and pH measurements. The rSAK-2 concentration was analyzed using RP-HPLC.

Final growth medium optimization and fine-tuning was performed in fed-batch mode. Following of above mentioned cultivation procedures, a feeding solution (FS) containing peptone and yeast extract as well as methanol and glycerol was added to the cultures batch-wise at a rate of 10 mL feed per 100 mL culture every 24 hours starting at 24 hours of fermentation. Flasks were shaken at 30°C and 180 rpm for a total of 96 hours. Samples were taken every 24 hours for OD_600 _and pH measurement. The rSAK-2 concentration was determined using RP-HPLC analysis.

#### Stirred bioreactors of 300 mL, 2 L, 8 L and 80 L scale

400 mL DASGIP parallel stirred tank bioreactors (DASGIP AG) with a maximum working volume of 300 mL were used for fed-batch process optimization, while a 3 L Biostat B-Plus, a 15 L Biostat C and a 150 L Biostat D (Sartorius Stedim Biotech) system with a maximum working volume of 2 L, 10 L and 100 L, respectively, were used for scale-up studies and protocol consistency trials. For all bioreactor studies, cells were grown in an in-house developed medium supplemented with 2% (w/v) glycerol as a carbon source. After sterilization, the medium was inoculated with an appropriate volume of an overnight grown shake flask YPG culture to reach an initial OD_600 _of 1.5 – 2.0. The pH was measured with a standard Ingold probe and controlled using either 42.5% (w/v) phosphoric acid solution or 17.3% (v/v) ammonium hydroxide solution. Foam formation was controlled manually using a 10% (w/w) Struktol solution.

The fermentation process was divided into a glycerol batch phase (19–22 hours) and methanol/glycerol fed-batch phase (24–25 or 49–51 hours). The period of fermentation was set to 70–73 hours. During the batch phase, cells continuously consume glycerol present in the growth medium. Depletion of the carbon source is signaled by a sharp increase in dissolved oxygen (DO) concentration. At the carbon source depletion signal, the medium was supplemented with a mixture of methanol/glycerol/peptone/yeast extract to induce expression of the heterologous protein. For the trials on a 300 ml scale, the air flow rate was 0.5 L/min with 500 rpm stirring speed. Due to the larger volume and the different geometry of the 2 L vessel, the air flow rate herein had to be adjusted to 2 L/min and the stirring speed to 800 rpm. Temperature was kept constant throughout the fermentation at 30°C.

Up to 3 samples were drawn from the fermenter at defined times daily for OD_600 _measurement. The rSAK-2 concentration was measured by RP-HPLC. In addition, final samples were subjected to further protein analysis using Western Blot. At the end of the fermentation, cells were pelleted by centrifugation at 4500 rpm for 10 minutes at 4°C. The cell pellet was discarded and the supernatant was subjected to sterile filtration and stored at −20°C for further downstream processing. Alternatively, at the 8 L and 80 L culture volumes, tangential flow filtration using a Sartocon Hydrosart (Sartorius Stedim Biotech) membrane with a 0.45μm cut-off was applied for cell-free supernatant harvest.

### Analytical methods

#### Optical density

Optical density (OD) measurement at an absorbance reading between 0.1 and 0.5 at 600nm wavelength was performed using an Ultrospec 6300 pro Visible/UV spectrophotometer (Amersham Biosciences).

#### Microscopic examination

Cells were examined for morphology and contamination using an oil immersion MEIJI light microscope with a total magnification of 1000-fold.

#### Protein analysis by RP-HPLC

All rSAK concentrations reported were measured by RP-HPLC using a LC – 2010C HT apparatus from Shimadzu. The culture broth was centrifuged for 10 minutes at 13200 rpm in a 5415R Centrifuge (Eppendorf AG) at 4°C to remove cells and cell debris. The clarified supernatant was then filtered using 0.22μm syringe driven filters (Millipore) before being subjected to an RP-HPLC method that uses an PLRP-S (5μm) column with gradient elution by 0.1% TFA in water and 0.1% TFA in acetonitrile as mobile phase. Non-glycosylated rSAK from *E. coli* (THR-174) served as reference and calibration material.

#### Protein analysis by Western blot

Expression clone selection: Samples were prepared by transferring 40 μL supernatant or cell-extract to 4x SAB buffer (reducing conditions) followed by heating for 5 min at 95°C in a thermoblock. Afterwards, samples of cell-extracts were centrifuged for 2 min at 13.000 rpm to remove cell debris. Samples were stored at −20°C. Loading volume was 7–21 μL per slot. SDS gel electrophoresis was executed with precast gels in a Criterion-Cell (BioRad). Voltage was adjusted to 150 V. The following precast gels were used for the expression studies: 4–20% Criterion precast gradient gel (BioRad #354-0032) in a Criterion-Cell (BioRad). Electroblotting was done using the BioRad Criterion blotter.

Fermentation screening: Supernatant samples were prepared in the same way as for RP-HPLC. Gel electrophoresis was performed using NuPAGE® Novex® Mini 12% Gels (Invitrogen) run at 200 volts for 40 minutes to separate proteins according to their molecular weight under non reduced conditions. Proteins from gels were electrophoretically transferred at 25 volts for 1 hour onto 0.2 μm nitrocellulose membrane. Immunodetection of rSAK was performed using a mouse monoclonal antibody developed against rSAK-1 (provided by ThromboGenics NV).

#### Plasminogen activation assay (PAA)

The activity of fermentation supernatant samples was compared with SAK reference standard (STAR: NIBSC catalogue number 94/718) as described in Collen et al. [25] with minor differences:

The assay is based on the activation of plasminogen to plasmin by SAK action. The resulting plasmin splits off enzymatically from a chromogenic tripeptide derivative p-nitroaniline. The kinetic course of this pigment release is observed photometrically at 405 nm using a UV spectrophotometer equipped with a heat-controlled cell holder. The measurement used is the rate of change of the optical density for each sample (in terms of slope Absorbance/second; A/sec). With each test series a standard curve is recorded (plot of concentration on x-axis and A/sec on y-axis) using SAK STAR 94/718. On the basis of this standard curve, the activity of the samples is established by reference to the curve by extrapolation of obtained A/sec for each sample to obtain its concentration. The reaction is performed by mixing of 5μL of SAK containing material with 78.5 μL 0.05 M sodium phosphate pH 7.4, 15 μL 10 μM human glu-Plasminogen (HTI) and 1.5 μL 1 μM human Plasmin (HTI), this mixture is incubated at 37°C for 5 minutes. 40 μl of the mixture is added to cuvette containing 960 μL of prewarmed 3mM substrate (S-2403, Chromogenix) inside the heat-controlled holder and measurement is directly started.

## Competing interests

The authors declare that they have no competing interests.

## Authors’ contributions

MM and MI carried out the fermentation runs in small scale and medium scale. MG carried out all analytical experiments. JR participated and carried out a critical manuscript revision for important intellectual content. SG and AA contributed all fermentation runs at large scale and helped to draft the manuscript. FK carried out all experiments in the microliter scale in the BioLector system. FM has drafted and revised the manuscript. All authors read and approved the final manuscript.
